# Molecular imaging of psoriatic arthritis

**DOI:** 10.1097/BOR.0000000000001098

**Published:** 2025-05-16

**Authors:** Sam Groothuizen, Conny Jacoba van der Laken

**Affiliations:** Department of Rheumatology & Clinical Immunology, Amsterdam Institute for Infection & Immunity, Amsterdam University Medical Centers, Amsterdam, The Netherlands

**Keywords:** diagnostics, molecular imaging, prediction, psoriatic arthritis, therapy monitoring

## Abstract

**Purpose of review:**

Psoriatic arthritis (PsA) is a chronic inflammatory disease associated with psoriasis. Conventional imaging techniques are used to diagnose the disease and detect long-term structural changes. This review will assess molecular imaging in PsA, to evaluate its potential additive value over conventional and advanced anatomical imaging methods (e.g. ultrasound and MRI).

**Recent findings:**

Current research is primarily focused on the molecular imaging technique PET/computed tomography (PET/CT) imaging, in which different tracers have been investigated. Fluorodeoxyglucose (FDG) can visualize disease activity and subclinical inflammation. New tracers targeting inflammatory sites have also been studied, such as FAPI (fibroblast activation protein inhibitor). Moreover, NaF (sodium fluoride) shows promise for imaging of new bone formation. Next to PET/CT, also fluorescence imaging and multispectral optoacoustic tomography have been investigated in the context of PsA.

**Summary:**

Molecular imaging techniques hold promise for early diagnosis, monitoring and management of PsA. Future research is needed to define the role of molecular imaging relative to conventional and anatomical imaging techniques in patient care.

## INTRODUCTION

Psoriatic arthritis (PsA) is a chronic inflammatory musculoskeletal condition affecting both peripheral joints and the axial skeleton. Up to 30% of psoriasis (PsO) patients develop PsA, and in 92% of PsA patients, the disease is preceded by psoriasis [1]. Early diagnosis is critical to prevent irreversible joint damage. Persistent inflammation leading to structural damage can significantly reduce the quality of life [2]. Diagnostic delay of more than 6 months is associated with the development of peripheral joint erosions and worse long-term physical function [3]. In addition, referral and diagnosis within 1 year are linked to improved clinical outcomes [4]. This highlights the importance of early diagnosis, in which imaging plays a critical role. Moreover, imaging may have additive value in accurate monitoring of disease activity and therapeutic efficacy. 

**Box 1:**
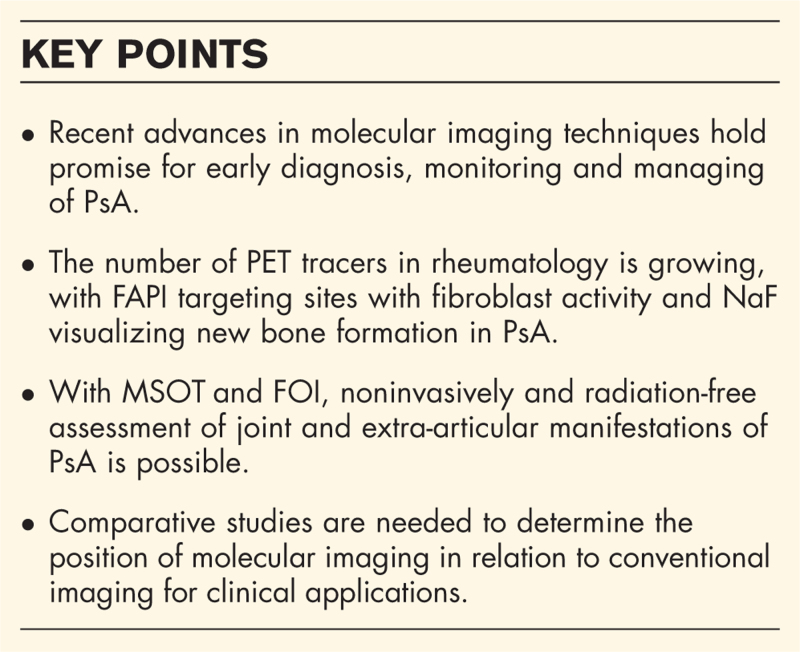
no caption available

Conventional imaging techniques have long been the standard in clinical practice. X-ray and computed tomography (CT) can visualize structural changes, but lack sensitivity for detecting early inflammation and do not provide information on soft tissues and actual disease activity. X-rays can only detect advanced damage and are not sensitive to early changes. Ultrasound is a widely available technique that visualizes inflammation in soft tissues in and around peripheral joints and entheses as well as surface pathology of the bones, but its ability to assess deep or axial structures is limited and bone pathology cannot be depicted. Additionally, high inter-observer variability reduces its reliability. Another mostly accessible imaging technique is magnetic resonance imaging (MRI), which can visualize inflammation and deep lesions, both in soft tissue and in the bone [5]. However, mostly limited fields of view are applied and its use is restricted by interference from metal implants.

Molecular imaging holds promise as an important addition to current imaging tools, since clinical examination alone may not reliably detect (sub)-clinical inflammation. Unlike above-mentioned imaging techniques, which primarily provide anatomical information, molecular imaging offers functional and molecular insights into tissues. It is often combined with anatomical imaging modalities, to anatomically reference molecular activity. Molecular imaging aims to add value for early detection of disease activity and monitoring of therapeutic efficacy [6]. This review will discuss molecular imaging techniques in PsA, from the early-stage bone scintigraphy and SPECT, to newer techniques such as positron emission tomography (PET) and fluorescence imaging. The utility of these modalities in PsA and their potential additive value over anatomical imaging techniques will be discussed. Table 1 summarizes an overview with the key features of these molecular imaging techniques.

**Table 1:** Overview with key features of molecular imaging techniques

	Radiation exposure	Advantages	Disadvantages	Whole body imaging
SPECT	Moderate	Widely available, combines metabolic and anatomical information. Option to combine with CT.	Lower resolution than PET and limited sensitivity.	Suitable, but often used for regional imaging
PET/CT	High	High sensitivity and spatial resolution, combines metabolic and anatomical information. Different tracers applicable.	Expensive, limited availability, radiation burden.	Suitable, commonly used for whole-body imaging
FOI	None	Noninvasive, high spatial resolution, real-time imaging.	Limited depth penetration.	Not suitable, imaging of smaller, localized regions
MSOT	None	Noninvasive, assess tissue composition, real-time imaging.	Limited depth penetration, not widely available.	Not suitable, imaging of smaller, localized regions

## BONE SCINTIGRAPHY AND SINGLE-PHOTON EMISSION COMPUTED TOMOGRAPHY

Bone scintigraphy was one of the earliest molecular imaging techniques explored in PsA. It involves the use of technetium-99m-labeled diphosphates, which are bound during the formation of calcium phosphate to osteoid surfaces at sites of active mineralization. Despite its ability to visualize joint involvement in PsA and subclinical involvement in PsO, bone scintigraphy lacks specificity [7]. As a result, MRI and ultrasound are more widely used in routine clinical practice. Other radiotracers have been investigated, but did not provide significant additional diagnostic value [8–11].

Single-photon emission computed tomography (SPECT) improves upon traditional bone scintigraphy by enabling three-dimensional reconstruction with higher spatial resolution. When combined with CT, areas with accumulated radiotracer can be enhanced with anatomical localization. An advantage of SPECT/CT is its applicability in patients with contraindications to MRI, such as those with metal implants. However, since MRI is noninvasive, carries no radiation burden, and offers superior soft tissue visualization, it remains more widely used in clinical practice [12]. Research on the use of SPECT in PsA is limited, though one study in spondyloarthritis patients demonstrated that SPECT/CT had better diagnostic accuracy compared to planar bone scanning in detecting sacroiliitis [13]. Currently, most research on novel radiotracers is focused on PET, as PET provides significantly higher sensitivity and spatial resolution and more detailed insights.

## POSITRON EMISSION TOMOGRAPHY (PET)

PET is a more advanced molecular imaging technology with a two to three- fold higher sensitivity than SPECT [14]. PET visualizes molecular tissue activity by detecting gamma photons emitted in opposite direction as a radiolabeled isotope decays. This technique allows for three-dimensional reconstruction of molecular expression and activity. Hybrid PET/CT or PET/MRI imaging offers the advantage of assessing the distribution of molecular activity along with anatomical localization throughout the entire body in a single scan. Various PET tracers can be used to identify inflammatory and molecular activity in bone contributing to specificity of imaging at molecular level.

## FDG (FLUORODEOXYGLUCOSE)

One of the most widely used PET tracers is 2-deoxy-2-[fluorine-18]fluorodeoxyglucose ([18F]-FDG), which accumulates in regions with high glucose metabolism, thereby visualizing overall metabolic activity. FDG PET has been studied in the context of many rheumatic diseases, including rheumatoid arthritis, spondyloarthritis and systemic disorders with arthropathy [15]. The largest limitation of FDG is its lack of specificity, since uptake can occur in both inflammatory and non-inflammatory tissues outside the joints [16].

Multiple studies have shown elevated FDG uptake in inflamed joints, tendons and entheses in patients with PsA. Notably, uptake was significantly higher in joints with synovitis compared to unaffected joints, with particularly strong uptake in the distal interphalangeal (DIP) joints and nail beds, which are characteristic PsA features [17,18]. Research has shown that whole-body joint assessment is even possible with use of ultra-low dose FDG PET [19]. Furthermore, subclinical inflammation has been detected in both joints and blood vessels in PsO patients without PsA [20]. Takata *et al.*[21] reported asymptomatic enthesitis on FDG PET/CT in six of 18 PsO patients, suggesting the potential of identifying early, subclinical PsA. These subclinical PsA patients had higher scalp and nail bed PsO involvement, which are known risk factors for developing PsA [22]. However, the clinical relevance of such subclinical findings remains uncertain, as most of these patients did not develop arthritis during follow-up. These findings emphasize the potential of FDG PET/CT for early disease detection, although the clinical implications of subclinical PsA remain uncertain.

Beyond musculoskeletal inflammation, FDG PET/CT has provided insights into systemic inflammation in PsA, particularly in the context of vascular involvement, a known comorbidity [23]. Studies have linked aortic metabolic activity to PsA, even after adjusting for cardiovascular risk factors, and have shown associations with metabolic dysregulation and coronary artery disease in biologic-naive patients [24,25]. Another study found increased aortic vascular inflammation in PsA, although not associated with other disease-related parameters [26^▪^].

Several studies have also evaluated changes in FDG uptake after treatment. For instance, a decrease in FDG uptake was observed after TNF-inhibitor therapy in ankylosing spondylitis (AS) and PsA patients, correlating with clinical improvement, although the results were not statistically significant in the small PsA subgroup (*n* = 8) [27]. In another cohort with six PsA and five PsO patients, FDG uptake decreased in both patient groups after TNF-inhibitor therapy [28]. These findings suggest that FDG PET could be of value for monitoring therapeutic effects. Additionally, a study comparing DMARD-naive and DMARD-failure patients found no significant differences in FDG uptake, suggesting that both groups could be combined in future PET/CT research [29^▪^].

Recent studies have also explored the effects of therapy on vascular inflammation in psoriatic disease. While one study showed a reduction in FDG uptake in the thoracic aorta after 6 months of biologic therapy, another study in PsO patients showed no changes in aortic vascular inflammation after apremilast therapy [30,31].

## NAF (SODIUM FLUORIDE)

[18F]Sodium Fluoride (NaF) is a bone-specific tracer that visualizes active osteoblastic-driven bone synthesis by binding to hydroxyapatite, forming fluorapatite at sites of molecular bone formation. Histological analysis of PET-guided bone biopsies of axSpA patients has shown increased osteoid formation and osteoblastic activity in areas with high NaF uptake, indicating its specificity of imaging new bone formation [32]. In PsA, NaF uptake may provide additive value to visualize new bone formation. [18F]NaF PET/CT offers superior image quality compared to traditional bone scintigraphy and is an ideal tracer due to its rapid bone uptake, low protein binding, fast blood clearance and high bone-to-soft-tissue background [33,34].

The first study using NaF PET/CT in PsA assessed the bone-enthesis-nail complex in DIP joint disease in PsA patients compared with osteoarthritis and healthy controls [35]. In 2019, an *Image in Rheumatology* was published demonstrating hypermetabolic activity in knee enthesitis in a PsA patient using NaF PET/MRI [36]. One-year follow-up of a PsA patient revealed decreased NaF uptake after 1 year of TNF-inhibitor treatment [37].

**FIGURE 1:**
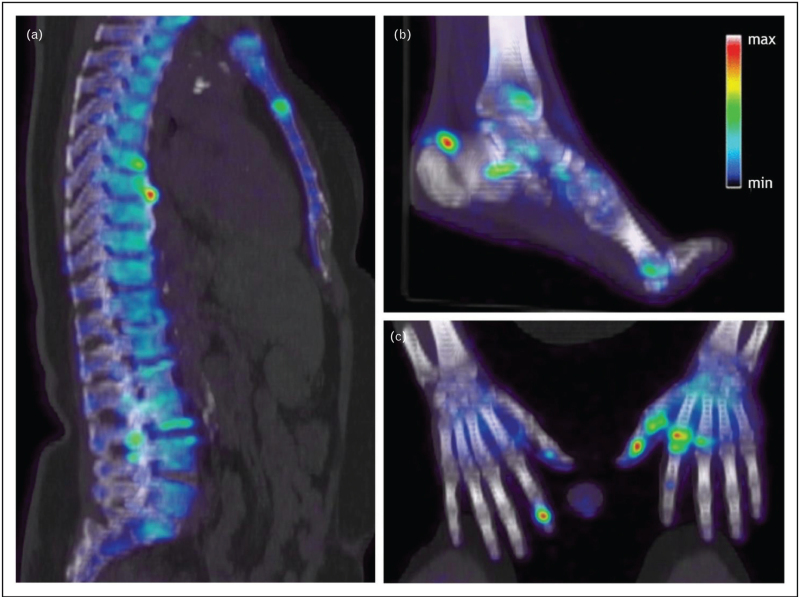
NaF uptake on PET/computed tomography in psoriatic arthritis patients. Figure 1 shows NaF enhancement in the axial spine (a), at the Achilles tendon insertion (b) and in the distal interphalangeal and metacarpophalangeal joints of the hands (c) in psoriatic arthritis patients.

In a cohort study investigating NaF PET/CT in PsA, 16 patients underwent whole-body imaging. Ten percent of evaluated joints and entheses showed increased NaF uptake. Notably, only 18% of PET-positive joints and 30% of PET-positive entheses were clinically symptomatic, indicating that NaF PET/CT can detect molecular new bone formation in both clinically symptomatic and asymptomatic lesions. The majority of PET-negative lesions were also clinically negative. Furthermore, in eleven patients, axial lesions were observed [38]. Figure 1 shows NaF uptake on PET/CT in PsA patients. Preliminary data also show changes in NaF uptake following TNF-inhibitor therapy after 12 weeks indicating a potential of early monitoring of therapeutic impact on new bone formation [39].

## FIBROBLAST ACTIVATION PROTEIN INHIBITOR (FAPI)

68Ga-FAPI-04 is a novel PET tracer that binds with high affinity to fibroblast activation protein (FAP), a cell surface marker upregulated in fibroblasts during tissue remodeling, such as present in inflammation [40]. Recent studies have reported FAPI uptake in PsO and PsA. A case report described FAPI uptake in the spine, feet and hip joints of a PsA patient [41]. In a cohort study, 36 PsO patients with arthralgia were evaluated for fibroblast activation in joints and entheses, of which 29 showed increased FAPI uptake. Eight percent of joints and 7% of entheses showed increased uptake, predominantly in large joints and the lower limbs. There was a significant relationship between FAPI signal intensity and the number of tender joints and entheses, though no correlation with ultrasound findings was observed. Importantly, patients with FAPI uptake had a higher risk of developing PsA, independent of ultrasound findings [42^▪▪^].

In a comparison study of [18F]NaF and 68Ga-FAPI PET/CT in 16 PsA patients, NaF showed uptake in all patients, while FAPI showed uptake in half of the patients. 277 joints showed NaF uptake and 71 showed FAPI uptake; 58 were positive on both scans. Interestingly, only FAPI positivity showed positive correlation with clinical features [43^▪^]. This suggests that FAPI may better reflect active inflammation than NaF, which detects new bone formation regardless of clinically active inflammatory symptoms.

Many other PET tracers have been studied in the field of rheumatology, mostly for imaging of rheumatoid arthritis [44]. The number of PET targets is growing, such as macrophage or stromal cell markers. The use of these tracers is still experimental, and larger PET studies of PsA patients are needed. But the growing number of identification of PET targets for different aspects of disease holds promise for improved disease detection and personalized treatment strategies.

More comparative studies with other imaging modalities are needed to define the best indication in future applicability of PET in PsA. While FDG can visualize disease activity and subclinical inflammation, its role in early diagnosis, cardiovascular risk stratification and therapeutic monitoring needs more investigation. Other tracers targeting inflammatory sites such as FAPI tracers are upcoming. Moreover, NaF shows promise for imaging of new bone formation already after several weeks, though more research is needed to define its role in evaluating disease activity and treatment response. Future research should focus on addressing the specificity of PET tracers, defining uptake cut-offs to distinguish arthritis from other non-inflammatory conditions, and validating tracers in larger cohorts of PsA patients and at-risk populations. The major limitations of PET, including availability, cost and radiation burden, are expected to improve with the increasing applicability of new technologies like total-body PET, which offers 20 times enhanced sensitivity and faster scanning [45].

## FLUORESCENCE OPTICAL IMAGING

Fluorescence optical imaging (FOI) has emerged as promising technique for visualizing (micro)circulation and vascular permeability of superficial lesions using an intravenous fluorescent contrast agent, typically indocyanine green (ICG), without ionizing radiation. It is particularly useful in PsA, as it effectively visualizes superficial structures like the hands, which are frequently affected. During the scan three phases can be distinguished: early (P1), intermediate (P2) and late (P3) enhancement of varying fluorescence intensities in the fingertips [46].

Studies have demonstrated FOI's sensitivity in detecting (subclinical) inflammation in the finger joints in PsA patients and PsO patients at risk of developing PsA [46–50]. Disease chronicity influenced fluorescence patterns, with suspected PsA cases showing more P1 enhancement, while confirmed PsA cases exhibited more P2/P3 enhancement [48]. Follow-up confirmed suspected PsA cases, with more findings in P3 in patients with progression [51].

Beyond joint imaging, FOI has proven useful in visualizing extra-articular disease manifestations. Wiemann *et al.*[52] introduced the “green nail” concept, a highly specific sign of impaired microcirculation of the nail bed, observed only in PsA patients. Furthermore, subclinical subdermal skin inflammation has been identified in both PsA and PsO patients [53]. Noteworthy, body weight influenced FOI results. FOI has also shown potential as a tool for therapeutic monitoring [54].

FOI is a noninvasive, radiation-free imaging technique that offers high spatial resolution and real-time imaging, making it useful for assessing both joint and extra-articular manifestations of PsA. However, its poor tissue penetration due to tissue scattering and light absorption prevent its application for whole-body imaging and only allow for detection of lesions at a depth of up to approximately 3 cm, although new developments may allow for detention up to 8 cm [55]. Therefore, for whole-body imaging and visualization of deeper lesions, PET remains superior. Standardized FOI nomenclature and an automated scoring method will facilitate more comprehensive future research [56,57].

## MULTISPECTRAL OPTOACOUSTIC TOMOGRAPHY

Multispectral optoacoustic tomography (MSOT) is a novel ultrasound technique that combines ultrasound imaging with optical illumination to noninvasively assess tissue composition, including oxygenation, collagen, lipid content and vascularization. It detects laser-induced ultrasound signals absorbed by tissue chromophores, such as (de)oxygenated hemoglobin, without ionizing radiation [58]. Like FOI, MSOT is limited to superficial lesions and unsuitable for whole body imaging.

In PsO patients, MSOT detected higher blood content and oxygenation of the finger joints compared to healthy controls, suggesting detection of early inflammation [59]. Additional studies explored specific tissue changes associated with PsA [60,61^▪^]. Although different findings were reported in entheses and joints, the results highlight the presence of immunometabolic tissue changes in psoriatic disease. In addition, an intra and inter-observed variability study confirmed the reproducibility of MSOT results, supporting its potential as a reliable, novel ultrasound technique [62]. Overall, MSOT holds potential for assessing tissue changes across different disease stages of PsA. For broader clinical applicability, standardized protocols will be required to enable consistent evaluation of individual findings.

## CONCLUSION

Molecular imaging techniques offer potential for early diagnosis, monitoring and managing of PsA. These modalities can provide novel molecular and pathophysiological insights into disease activity, progression, extra-articular manifestations and treatment effects. Future research should focus on standardizing these imaging techniques and quantification of the data as well as refining diagnostic thresholds for more comprehensive future research. New developments in artificial intelligence may facilitate further future implementation. The determination of the position of molecular imaging techniques in relation to conventional and advanced anatomical imaging techniques is essential for clinical applications, therefore comparative studies are needed. Development of imaging strategies making use of the new technical developments will improve early detection, facilitate personalized treatment and ultimately enhance long-term outcomes in PsA.

## Acknowledgements

*None.*

### Financial support and sponsorship

*None.*

### Conflicts of interest

*There are no conflicts of interest.*

## REFERENCES AND RECOMMENDED READING

Papers of particular interest, published within the annual period of review, have been highlighted as:

▪ of special interest
▪▪ of outstanding interest
